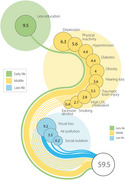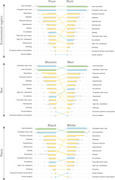# The potential for dementia prevention in Brazil: an updated population attributable fraction calculation for 14 modifiable risk factors

**DOI:** 10.1002/alz70860_105891

**Published:** 2025-12-23

**Authors:** Claudia Kimie Suemoto, Wyllians Vendramini Borelli, Ismael Luis Calandri, Laiss Bertola, Raphael Machado Castilhos, Paulo Caramelli, Ricardo Nitrini, Sonia Brucki, Jerson Laks, Naheed Mukadam, Gill Livingston, Cleusa P Ferri

**Affiliations:** ^1^ Division of Geriatrics, University of São Paulo Medical School, São Paulo, São Paulo, Brazil; ^2^ Universidade Federal do Rio Grande do Sul, Porto Alegre, Rio Grande do Sul, Brazil; ^3^ Centro de Memória, Hospital Moinhos de Vento, Porto Alegre, RS, Brazil; ^4^ Clinical Hospital of Porto Alegre, Porto Alegre, Rio Grande do Sul, Brazil; ^5^ Brain Institute of Rio Grande do Sul (InsCer), PUCRS, Porto Alegre, Rio Grande do Sul, Brazil; ^6^ Fleni, Buenos Aires, Buenos Aires, Argentina; ^7^ Alzheimer center, VUMC, Amsterdam, Netherlands; ^8^ University of Sao Paulo Medical School, Sao Paulo, Sao Paulo, Brazil; ^9^ Hospital de Clínicas de Porto Alegre, Porto Alegre, Rio Grande do Sul, Brazil; ^10^ Universidade Federal de Minas Gerais, Belo Horizonte, MG, Brazil; ^11^ Cognitive and Behavioral Neurology Unit ‐ University of São Paulo, São Paulo, Brazil; ^12^ University of São Paulo Medical School, São Paulo, Brazil; ^13^ Federal University of Rio de Janeiro, Rio de Janeiro, Brazil; ^14^ University College London, London, London, United Kingdom; ^15^ Hospital Alemão Oswaldo Cruz, São Paulo, São Paulo, Brazil; ^16^ Universidade Federal de São Paulo (UNIFESP), São Paulo, São Paulo/SP, Brazil

## Abstract

**Background:**

The Lancet Commission on dementia prevention, intervention, and care 2024 updated the list of modifiable risk factors to include 14 factors. The potential for dementia prevention seems to be greater in low‐ and middle‐income countries (LMIC) due to the higher prevalence of these factors. This study aims to provide the first LMIC figure for the potential for dementia prevention in Brazil attributed to 14 modifiable risk factors.

**Method:**

Data was retrieved from 9,949 participants aged 50 years or older from the nationally representative second wave of the Brazilian Longitudinal Study of Aging (ELSI‐Brazil) conducted between 2019 and 2021. The prevalence of modifiable risk factors was estimated, and principal component analysis was used to account for factor communalities. Overall and individual population attributable fractions (PAF) were calculated using relative risks from the 2024 Lancet Commission report. Stratified analyses by sex, race, and macro regions were performed to assess disparities in dementia risk.

**Result:**

The overall PAF for the 14 modifiable risk factors was 59.5% (95% CI=58.5‐60.5). The three risk factors with the highest PAFs were less education (9.5%, 95% CI=8.9‐10.1), untreated visual loss (9.2%, 95% CI=8.6‐9.8), and midlife depression (6.3%, 95% CI=5.8–6.8). The overall PAF was similar across race and region but was higher among women (61.1%, 95% CI=59.9‐62.4) compared to men (58.2%, 95% CI=56.7‐59.8).

**Conclusion:**

Almost 60% of dementia cases in Brazil could potentially be prevented by addressing 14 modifiable risk factors. Sex‐specific tailored public health strategies could further reduce the dementia burden in Brazil.